# Physiological Intracellular Crowdedness is Defined by the Perimeter-to-Area Ratio of Sub-Cellular Compartments

**DOI:** 10.3389/fphys.2012.00293

**Published:** 2012-07-23

**Authors:** Noriko Hiroi, Takahiro Okuhara, Takeshi Kubojima, Keisuke Iba, Akito Tabira, Shuji Yamashita, Yasunori Okada, Tetsuya J. Kobayashi, Akira Funahashi

**Affiliations:** ^1^Department of Bioscience and Informatics, School of Fundamental Science and Technology, Keio UniversityYokohama, Japan; ^2^Department of Pathology, School of Medicine, Keio UniversityTokyo, Japan; ^3^Institute of Industrial Science, The University of TokyoTokyo, Japan

**Keywords:** fractal dimension, percolation, molecular crowding, mean square displacement, surface-to-volume ratio

## Abstract

The intracellular environment is known to be a crowded and inhomogeneous space. Such an *in vivo* environment differs from a well-diluted, homogeneous environment for biochemical reactions. However, the effects of both crowdedness and the inhomogeneity of environment on the behavior of a mobile particle have not yet been investigated sufficiently. As described in this paper, we constructed artificial reaction spaces with fractal models, which are assumed to be non-reactive solid obstacles in a reaction space with crevices that function as operating ranges for mobile particles threading the space. Because of the homogeneity of the structures of artificial reaction spaces, the models succeeded in reproducing the physiological fractal dimension of solid structures with a smaller number of non-reactive obstacles than in the physiological condition. This incomplete compatibility was mitigated when we chose a suitable condition of a perimeter-to-area ratio of the operating range to our model. Our results also show that a simulation space is partitioned into convenient reaction compartments as an *in vivo* environment with the exact amount of solid structures estimated from TEM images. The characteristics of these compartments engender larger mean square displacement of a mobile particle than that of particles in smaller compartments. Subsequently, the particles start to show confined particle-like behavior. These results are compatible with our previously presented results, which predicted that a physiological environment would produce quick response and slow exhaustion reactions.

## 1 Introduction

The intracellular environment is highly crowded with sub-cellular components. Those organella and polymers can be visualized using electron microscopic techniques. In our previous work, we demonstrated the crowding level and the characteristics of the intracellular environment based on investigation of the fractal dimension (*d_f_*) of transmission electron microscope (TEM) images (Hiroi et al., 2011a,b). At the same time, we explained the effect of the *in vivo* environment to the molecular behaviors following the transformation of the properties of biochemical reaction processes. The reaction process exhibits a quick response to the initial stimuli and slow exhaustion after a long reaction time, which might be the cause of the robustness of living matter. We also showed that if NRO exist as a large, smooth cluster in the reaction space, then those NRO do not affect the behavior of a mobile particle in the same way with randomly spread NRO. Nevertheless, the total volumes in those two conditions are equal.

We planned our simulation tests for this study based on the results presented above. If an *in vivo*-like environment can be reconstructed for a mobile particle by building solid structures with fractal models for which we found similarity with physiological solid structures, then the effects on the mobile particles can be confirmed.

The amount of solid obstacles in an intracellular environment is known to be the same as the volume of water (Fulton, 1982). It is nearly the threshold value of percolation in three-dimensional space. Percolation means that a mobile object can reach from one side to the other side of a space. As described in this paper, we investigated particular fractal models that consist of close volume of non-reactive obstacles (NRO) in reaction space to the percolation threshold (*p_c_*). The fractal models we used were the modified diffusion limited aggregation (DLA) model (Tolman and Meakin, 1989) and the cluster–cluster aggregation (CCA) model (Meakin and Djordjevi, 1985). We designated our modified DLA model as random DLA (rDLA). We produced another model by putting a single non-reactive obstacle randomly at each step of organization in a reaction space (random NRO, rNRO).

The DLA model expresses the process of aggregating Brownian particles. Later results showed that this model can represent the process of the colony formation of bacteria (Yamazaki et al., 2002). The particle aggregation process is defined as a diffusion-dependent process, i.e., the binding probability equals 1. Consequently, the progress of the organization depends on the diffusion probability, which is far less than 1.

The particular characteristics of CCA model compared with the other two models described above is that the NRO in this model moves after they collide and bind between two or among more particles. Diffusion-limited CCA and reaction-limited CCA exist. We assumed that polymerization of biomolecules in an intracellular environment might undergo a diffusion-limited process.

In our previous work, the outline of solid structures showed similar *d_f_* with that of the DLA model (Tolman and Meakin, 1989). The operating range for a mobile particle showed similar *d_f_* to that of the invasion percolation (IP) model (Wilkinson and Willemsen, 1983). To represent the shape of NRO clusters, we built rNRO, rDLA, and CCA. These models produce a connected pattern of NRO according to the respective algorithms. Each step of organizing NRO clusters was defined randomly in all models. As a result, the clusters produced by these models grow equivalently in all directions.

We examined whether an *in vivo*-like environment can be reconstructed for a mobile particle with these models. We calculated *d_f_* to assess the similarity between the artificial space and the physiological condition. We also calculated the mean square displacement (MSD) and diffusion constant to confirm that the behaviors of mobile particles in those environments are affected by the environments. Among our models, rNRO is constructed using single obstacle based processes. It follows that each obstacle moves independently and that it does not change the behavior depending on whether it is next to the other obstacles or not. rDLA is also constructed with single obstacle based processes. Once an obstacle is left in the next pixel of the other one, they are estimated as a new NRO cluster in this model, and are stopped from moving after the subsequent organizing step. The NRO clusters in CCA models grow until all the NRO are connected (completed CCA models). We stopped at several growing processes of CCA models and produced independent reaction space models to investigate the effects of the perimeter-to-area ratio of the operating range.

Our results demonstrated that environments that consist of single random walkers and clustered random walkers show different *d_f_*, different size distribution of NRO clusters and different effects to a mobile molecule in the operating range. The results also suggest that the distribution of the sizes and the shapes of the clusters are the keys of the *in vivo* environment, which is represented by the value of the perimeter-to-area ratio of the operating range.

## 2 Materials and Methods

### 2.1 Implementation of fractal model simulators

We implemented the following simulators with reconstructed reaction spaces that are characterized respectively by fractal models. We investigated the *d_f_* of the reconstructed reaction spaces and the distribution of the cluster size. We also investigated the effect on a diffusive molecule in the reaction space by calculating the mean square displacement (<*r*^2^>, MSD). To compare these parameters with results from physiological conditions, we investigated the *d_f_* of the TEM imaged space and the MSD of a mobile molecule in the operating range in TEM images.

#### 2.1.1 Reaction space with diffusion limited aggregation (DLA) model

We implemented a DLA model in our reaction space. The conditions of implementation are the following.

1. Displacement from the core and the farthest point in the cluster is *r_max_*.
2. The radius of the launching circle, *r_L.C._*, which is the starting line of Brownian particles, is defined as
  $$\begin{array}{rcll} r_{L.C.}=r_{max}+5 & & & \end{array}$$
3. The radius of the killing circle, *r_K.C._*, which is the line to force an end to tracking for a particle that has gone too far is defined as
  $$\begin{array}{rcll} r_{K.C.}=3r_{max} & & & \end{array}$$
  as it was defined in an earlier report (Yamazaki et al., 2002).
4. The simulation space is 10,000 × 10,000 square lattice.
5. The DLA core was put at the center of the square lattice.
6. A Brownian particle starts a random walk from a point on the circumference of L.C., which has a DLA core as its center.
7. When the Brownian particle reached the next lattice of DLA core or a DLA cluster, the particle stopped its random walk and the *r_max_* was recorded.
8. When the Brownian particle reached the K.C., the particle stopped its random walk and was removed from the simulation space.
9. Repeat processes 6, 7, and 8 for 10^5^ iterations.

**Figure 1:**
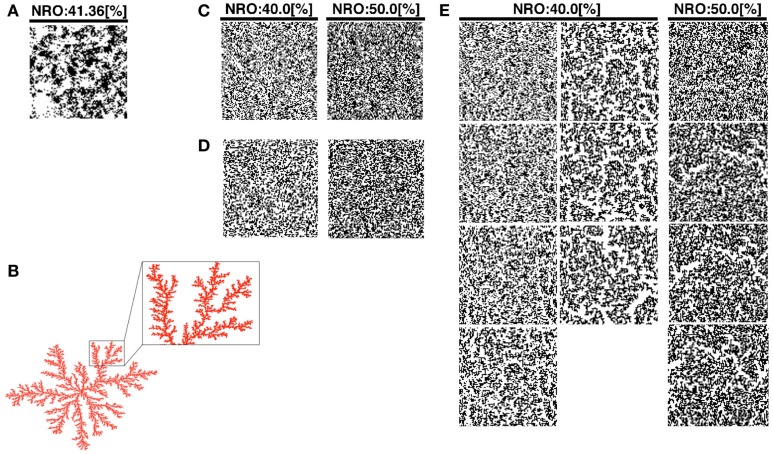
**Reconstructed reaction spaces for a mobile particle: (A), TEM images modified the size to 100 × 100 pixels were used as representative environment of physiological conditions**. **(B)** Example of simulated DLA model. Some branches of the structure were expanded to show the scale-free structure in the balloon. **(C)** Simulated structure of random NRO. The structure in the left panel consists of 40% of NRO. The structure in the right panel consists of 50% of NRO. **(D)** Simulated structure of random DLA. The structure in the left panel consists of 40% of NRO. The structure in the right panel consists of 50% of NRO. **(E)** The simulated structure of CCA. The left two lanes of panels consist of 40% of NRO. Panels in the lanes respectively show the structure at the 100th step, 300th step, 500th step, 700th step, 900th step, 1,000th step, and 2,000th step of each simulation. The right lane panels consist of 50% of NRO. Each panel in the lane respectively shows the structure at the 100th step, 300th step, 500th step, and 700th step.

The DLA model size shown in Figure 1B was produced by a smaller simulation space (1,500 × 1,500) than the one we used for our additional simulation.

#### 2.1.2 Reaction space with random DLA (rDLA) model

The DLA models need a core and L.C. for each Brownian particle (Tolman and Meakin, 1989). Consequently, the final cluster number is one. Also only one Brownian particle exists for each timing. This result is divergent from that obtained in a realistic intracellular environment. Therefore, we designed an original model designated as random DLA (rDLA). Details of the model are the following:

1. The simulation space is a 100 × 100 square lattice.
2. NRO were sprinkled in the lattice. The amount of the NRO was varied from 40 to 50% per the lattice size (=100 × 100).
3. A list of particles that are independent from any other particle was produced.
4. Particles were chosen from the list to allow movement of one step randomly among four directions on the lattice.
5. Repeat 3 and 4 for all the listed particles.

#### 2.1.3 Reaction space with a cluster–cluster aggregation (CCA) model

The CCA model was described as follows (Meakin and Djordjevi, 1985).

1. The simulation space is a 100 × 100 square lattice.
2. NRO were sprinkled in the lattice. The amount of the NRO was varied from 40 to 50% per lattice size (=100 × 100).
3. Numbering the clusters of NRO.
4. Choose one cluster and let it move one step among the four directions.
5. Repeat 3 and 4.

We produced histograms of the cluster size distribution based on investigation of the results of these models.

### 2.2 Cell culture

Cell culture reagents for 3Y1 cells were obtained from Wako Pure Chemical Industries, Ltd. (Japan). The cell lines were cultured routinely in Dulbecco’s Minimal Essential Medium (DMEM) supplemented with 10% fetal bovine serum (FBS) in a 5% CO_2_ incubator. We obtained a 3Y1 cell line from the Japanese Collection of Research Bioresources (JCRB) Cell Bank.

### 2.3 Transmission electron microscopy (TEM) images

#### 2.3.1 Imaging procedures by TEM

We obtained 165 images of rat fibroblast 3Y1 cells and analyzed them using box counting methods.

Source images were the subregion of the images of cytoplasmic region. Cells were collected on the day when the cells reached at the confluent condition to obtain the homogeneous population in their cell cycle (G1–G0 cells).

In preparation for TEM, the cells were fixed with 4% formaldehyde and 2% glutaraldehyde in 0.1 M phosphate buffer (pH 7.4) for 16 h at 4°C, and were fixed successively with 1% osmium tetraoxide in 0.1 M phosphate buffer (pH 7.4). Cells were dehydrated in graded ethanol and embedded in epoxy resin. Ultrathin sections (~60 nm thick) were prepared with a diamond knife and were electron-stained with uranyl acetate and lead citrate. They were examined using an electron microscope (H-7650; Hitachi, Ltd.).

#### 2.3.2 Calculation process of the fractal dimension (*d_f_*)

Parameter *d_f_* was derived from box counting as explained below (Chhabra et al., 1989).

First, the TEM images were binarized into objects and background using the auto-thresholding function of ImageJ (http://rsbweb.nih.gov/ij/). Briefly, this algorithm computes the average intensity of the pixels below or above a particular threshold. It then computes the average of these two values, increments the threshold, and iterates the process until the threshold is larger than the composite average. That is,

$$\text{threshold}=\frac{\left(\text{average}\;\text{background}+\text{average}\;\text{objects}\right)}{2}.$$

A scaling rule for the relation between the count and box size in box counting, assuming that these correspond respectively to details or the number of parts (*N*) and scale (ε) is given as

$$d_{f}=\underset{\text{ε}\to \infty }{\lim}\frac{logN_{\text{ε}}}{log\text{ε}},$$

where the limit is found as the slope of the regression line.

## 3 Results

### 3.1 Reconstruction of artificial reaction space models and their fractal dimensions

Earlier studies by the authors (Hiroi et al., 2011a,b) analyzed the *d_f_* of a physiological environment using TEM images. Results show that the *d_f_* values of physiological NRO outline and the operating range for a mobile particle were close to particular fractal models: the DLA model and the IP model. We found that a cluster–cluster aggregation model (CCA) is a better candidate as a representative model for the physiological environment when we consider the effect of noises or modifications dependent on the variation threshold definition.

Now, we prepared multiple DLA and CCA models to use them as the simulation space for a mobile particle in a crowded environment. First, we analyzed their *d_f_* values to compare the value of TEM images.

We produced a normal DLA structure (Figure 1B), random NRO structure (rNRO, Figure 1C), random DLA structure (rDLA, Figure 1D), and CCA (Figure 1E). We constructed rDLA and CCA models with 40–50% NRO, and an exception case (26%). At the same time, we prepared TEM image series which keep the same image size with artificial reaction spaces, to use as the control of the physiological condition. The basis to determine the range of the relative amount of NRO is the percolation threshold (*p_c_*) in two-dimensional space (*p_c_* = 0.593). The value denotes the relative size of the free space without obstacles in our case. Therefore, 40.7% of the entire reaction space is occupied by NRO in the condition of *p_c_*. When the relative amount of NRO is smaller than 0.407, a mobile particle, which is in the free space, can percolate from a side to the opposing side. Especially for CCA, we respectively stopped the organization process at the 100th, 300th (s300), 500th (s500), 700th (s700), 1,000th (s1,000), and 2,000th (s2,000) steps, and used the simulation space of a mobile particle. We can observe the self-organizing processes of NRO in these artificial models (Movie S1 in Supplementary Material).

**Figure A1:**
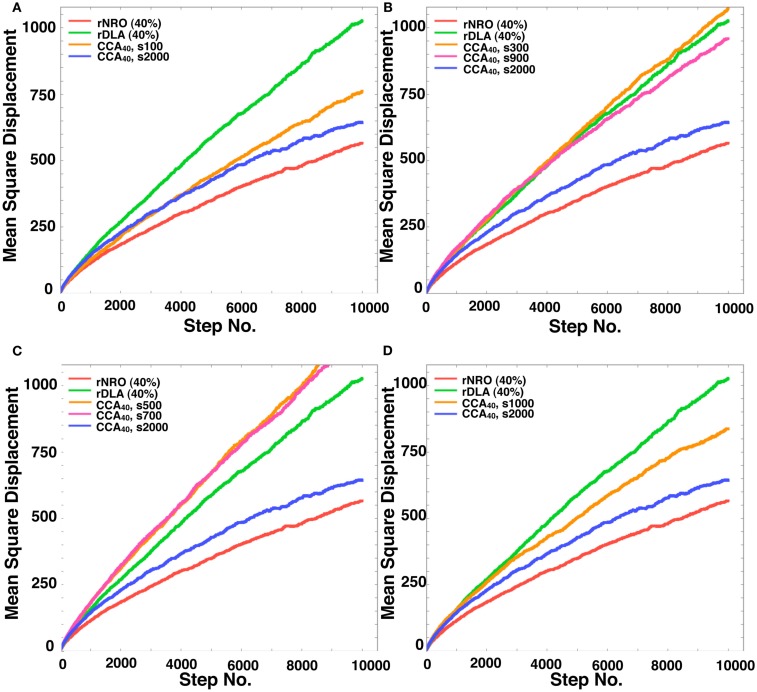
**Mean Square displacement of a mobile particle in each model**. **(A)** rNRO (40%), solid red line; rDLA (40%), solid green line; CCA_40_, s2000 (completed CCA), solid blue line; CCA_40_, s100 (preliminary CCA), solid orange line. **(B)** rNRO (40%), solid red line; rDLA(40%), solid green line; CCA_40_, s2000 (completed CCA), solid blue line; CCA_40_, s300 (early CCA), solid orange line; CCA_40_, s900 (completed CCA), solid magenta line. **(C)** rNRO (40%), solid red line; rDLA (40%), solid green line; CCA_40_, s2000 (completed CCA), solid blue line; CCA_40_, s500 (early CCA), solid orange line; CCA_40_, s700 (early CCA), solid magenta line. **(D)** rNRO (40%), solid red line; rDLA(40%), solid green line; CCA_40_, s2000 (completed CCA), solid blue line; CCA_40_, s1000 (completed CCA), solid orange line.

Here, we define the names of CCA models at the specific steps of their organization. The CCA with 40% of NRO (CCA_40_) organized until the 100th step, we designated as preliminary CCA. The CCA organized in the 300th to 700th step, we designated as early CCA. Over 900th step of simulated CCA we designated as completed CCA. The reason to categorize CCA dependent on the organizing steps was the difference of the effects on MSD of mobile particles among these models. By comparison with the growth of MSD of rDLA model with the same relative amount of NRO (40% in this case), s100, s900 and larger show slower growth, s300–s700 show faster growth of MSD during the simulation time (Figure A1 in Appendix). This property is completely different when the relative amount of NRO in a reaction space is greater than *p_c_*. In our case, no test in the reaction spaces with 50% NRO show a distinguishable case that is dependent on the organizing steps under this condition (Figures 7A,B). This difference between the results of CCA_40_ and CCA_50_ raised the prospect to us that we might find an important mechanism to bring the effect of environment to the molecular behaviors by analyzing these cases independently. Therefore, we defined preliminary CCA, early CCA, and completed CCA and distinguished the simulation results of those cases.

**Figure 2:**
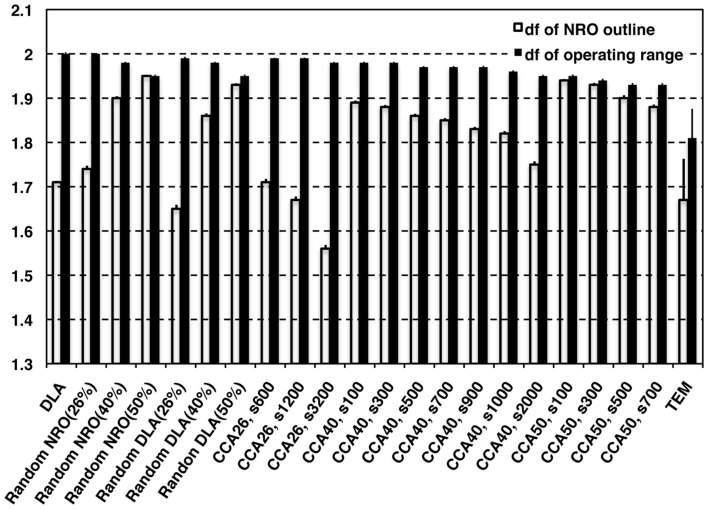
**Fractal dimensions of physiological environment and simulated models**. White bars represent the *d_f_* values of outline of NRO in models. Black bars represent the *d_f_* values of the operating range for a mobile particle in the models.

Figure 2 shows the *d_f_* of the outline of NRO clusters (white bars) and the *d_f_* of operating range for a mobile particle (black bars), which appears as the space without NRO. The TEM images were processed for data size to compare the characteristics directly with artificial reaction space models. The data size was uniform with the size of artificial reaction spaces (100 pixels × 100 pixels). The resolution of original TEM images was better before processing. Results show that the *d_f_* values obtainned in this study were slightly smaller than those obtained in our previous work (outline *d_f_* = 1.75, operating range *d_f_* = 1.81; Hiroi et al., 2011a,b). The outline *d_f_* of solid structures in TEM images was 1.67 ± 0.093 in this analysis. However, all the *d_f_* values of NRO outline of our fractal models appeared as the larger values than the *d_f_* of TEM images when the relative NRO amount was 40 or 50%, which is close to the amount with physiological conditions (41.36%) derived from the black area of binarized TEM images. Among these conditions, DLA (40%; *d_f_* = 1.71 ± 0.0032), CCA_40_, s2,000 (*d_f_ *= 1.75 + 0.0071) showed a value close to physiological *d_f_*. Results show that when the relative NRO amount is less than the physiological condition (26%), the *d_f_* values of our fractal models appeared as values close to the physiological condition [rNRO (26%): 1.74 ± 0.0074, rDLA(26%): 1.65 ± 0.0091, CCA_26_, s600: 1.71 ± 0.0075, s1,200: 1.67 ± 0.0077, s3,200: 1.56 ± 0.0081]. For *d_f_* values of the operating range of a mobile particle, we were unable to find any model that can represent a similar condition to that of the physiological environment.

These results clarified that it is possible to find a condition to reconstruct a fractal structure of NRO. However, no candidate represents the operating range for a mobile particle among our models.

To ascertain why no model can reconstruct the characteristics of the operating range, we examined the difference between physiological structures in TEM images and our artificial reaction spaces. Results show that the NRO clusters in the physiological environment have wide distribution in their size and shape. Nevertheless, the NRO clusters in artificial reaction spaces maintain a homogeneous shape, even the distribution of the size seems limited.

Our artificial models (DLA, rNRO, rDLA, and CCA) are filled with fine fiber-like or branch-like clusters (Figures 1B–E) by comparison with the physiological condition presented in Figure 1A. However, the physiological environment presented in Figure 1A involves both branch-like clusters and circular clusters. This difference between the characteristics of physiological solid structures and artificial NRO clusters might be the cause of the difficulty in reconstructing the characteristics of the operating range by models. Furthermore, the difference is potentially effective for the behaviors of a mobile particle in such environments. Moreover, the difference of the shape directly is related to the difference of perimeter-to-area ratio of clusters. The observed difference between artificial NRO clusters and the physiological solid structures are that physiological solid structures include circular clusters. The perimeter-to-area ratio of circles is smaller than the ratio of rectangles when those are the same square measure, especially when the narrow-side to long-side ratio of the rectangle is large, such as it is with a fiber-like or branch-like shape.

Based on the observation described above, we first quantify the distribution of the NRO cluster size and operating range for a mobile particle of each model to ascertain the critical factors of the cause of failure in the characteristics of the NRO clusters themselves, and to investigate the concrete relation between those factors to elucidate the effects on the behavior of a mobile particle.

### 3.2 Size distribution of NRO clusters

**Figure 3:**
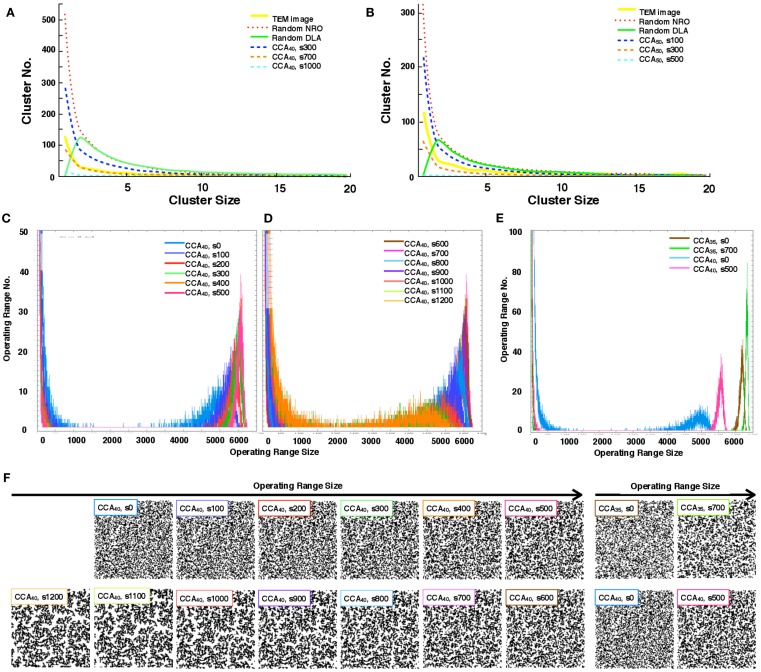
**Size distribution of the discrete clusters in each model**. **(A)** Size distribution of the discrete clusters in physiological conditions (TEM, yellow solid line), and all models with 40% NRO (Random NRO, red dots; Random DLA, green solid line, CCA at the 300th step, blue dashed line; CCA at the 700th step, orange dashed line: CCA at the 1,000th step, cyan dashed line). **(B)** Size distribution of discrete clusters in physiological condition (TEM, yellow solid line), and all models with 50% NRO (Random NRO, red dots; Random DLA, green solid line, CCA at the 300th step, blue dashed line; CCA at the 700th step, orange dashed line: CCA at the 1,000th step, cyan dashed line). **(C)** Size distribution of the operating range in the CCA model with 40% NRO (0 step, blue; 100th step, bluish purple; 200th step, red; 300th step, green; 400th step, orange; 500th step, pink). **(D)** Size distribution of operating range in CCA model with 40% NRO (600th step, brown; 700th step, magenta; 800th step, light blue; 900th step, purple; 1,000th step, deep orange; 1,100th step, light green; 1,200th step, light orange). **(E)** Size distribution of operating range in the CCA model with 35 or 40% NRO (CCA with 35% NRO at the 0th step, brown; CCA with 35% NRO at the 700th step, green; CCA with 40% NRO at the 0th step, light blue; CCA with 40% NRO at the 500th step). **(F)** Reaction space appearance at each condition. Images of corresponding simulated reaction spaces to the lines in panels **(C,D,E)** are captured and shown according to the peak value of the size of the distribution of their operating range. Each simulation condition is shown at the top-left position of each image. Six images arranged at the upper left space portray the appearance of reaction spaces used for the graph in **(C)**. Seven images arranged at the lower left space are the appearance of reaction spaces used for the graph in **(D)**. Four images in the right space are the appearance of reaction space used for the graph in **(E)**.

We quantify the distribution of the cluster size of each model. With the condition of 40 or 50% of NRO relative volume in the space, we found that the pattern of the size distribution of clusters in rNRO and all CCA models are similar (Figures 3A,B), but rDLA differs: it has a peak on the size 2 cluster. This result occurs because of the algorithm used to build rDLA (see Materials and Methods). However, rNRO and CCA models show their peak at size 1 (Figures 3A,B).

**Figure A2:**
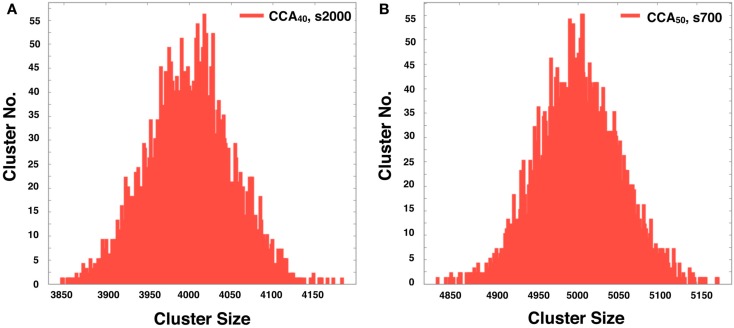
**The histogram shows the distribution of the NRO cluster size in 5,000 of completed CCA40, s2000 (A) and completed CCA50, s700 (B)**. The peaks of the respective histograms equal the theoretically derived value of each model (4,000 was the peak of the histogram of CCA_40_; 5,000 was the peak of the histogram of CCA_50_).

The CCA models show a specific feature in the large clusters. Generally, all NRO in CCA models aggregate as one cluster after a long simulation time. Consequently, the model has its peak of cluster size at the volume of NRO in the reaction space. Some examples are that if the case of NRO 40% in 100 × 100 lattice, then the peak will be 4,000. Also if the case of NRO 50% in 100 × 100 lattice, then the peak will be 5,000 (Figure A2 in Appendix). This result guarantees that the simulation was performed properly and that it produced correct results.

By comparison with the size distribution of NRO clusters in TEM images, the pattern of the cluster size distribution was confirmed to be close to that of the CCA model, especially the pattern organized until 700th step with 40% of NRO (CCA_40_, s700). This result demonstrates that one of our models (CCA_40_, s700, one of the early CCA) can reproduce the same size distribution of NRO cluster as that of the physiological condition. Another point suggested by the result is that the realistic solid structures in a cell appearing in TEM images do not consist of one connected aggregation. The completed CCA, in which all NRO are connected each other, cannot reproduce a similar size distribution of NRO clusters.

The distribution pattern of the cluster size was similar between CCA_40_ with a real cell. Therefore, we investigated more about the transition of operating range distribution of CCA_40_ (Figures 3C–E).

Histograms in Figures 3C,D show a particular pattern of the operation range size distribution which appears as the small peak in the range that was logically estimated above. Here the operation range is the free space for a mobile particle, which means that when the relative amount of NRO is 40% of the whole simulation space, the operation range is the remaining 60% of the whole space. The peak appears between 4,000 and 5,000 in the preliminary CCA. However, the major size of the operation range grew to be nearly 6,000 among early CCA models. For completed CCA, the peak of the operating range size was reduced until it became the same range with preliminary CCA. This tendency is visible in the different CCA, which consists of different relative amounts of NRO. Figure 3E shows the case of CCA_35_. Reaction space appearance at each condition is indicated in Figure 3F, in order to show the correlation of structural characteristics and the distribution of operating ranges. Images of corresponding simulated reaction spaces to the lines in Figures 3C–E are captured and shown according to the peak value of the size of the distribution of their operating range. The early CCA conditions which indicated a sharp peak at the largest size of operating range (CCA40 s300–s800, CCA35 s700) produced similar appearance of their reaction spaces, nevertheless both preliminary CCA and completed CCA showed extremely fine and homogeneously or coarse and inhomogeneously organized reaction spaces.

**Figure 4:**
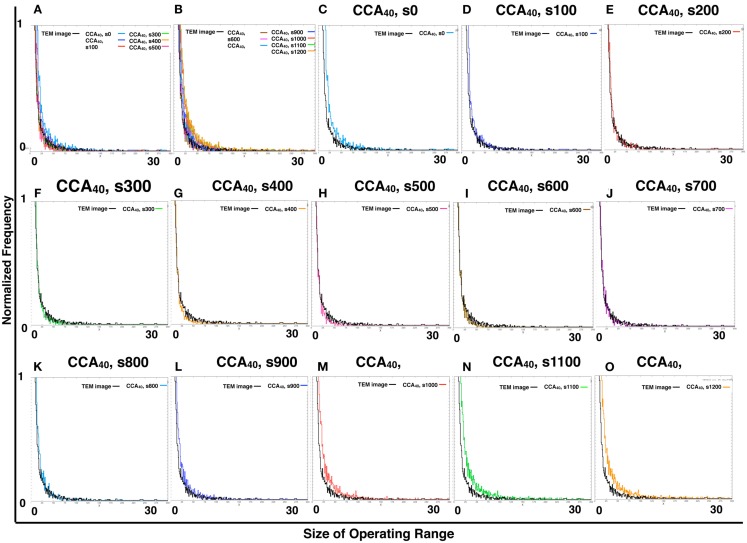
**Histograms of size distributions of operating ranges of TEM images and simulated reaction spaces**. Histograms in Figure 4 were produced from 161 TEM images using 161 images of CCA models with each condition to normalize both with the same procedure. The results are shown within the range of 0–300 at the *x*-axis for clear comparison between the distribution curve produced with TEM and the curve of our simulated reaction space. **(A,B)** Show the CCA_40_, s0–s500, and s600–s1200, respectively, and the size distribution of the operating range of TEM images (black lines). *x*-Axes show the size of the operating range (0–300). *y*-Axes show the normalized frequency (0–1). **(C–O)** Show only two lines in one panel: the size distribution of operating range of one of the model among CCA_40_, s0–s1200, and a TEM image. Results in **(C–E)** show the cases of preliminary CCA. Results in **(F–K)** show the cases of early CCA. The results in **(L–O)** show the cases of completed CCA.

By comparing the distribution curve of the size of operating range in our CCA models with TEM images (Figure 4), both the distribution curve of the preliminal (s0–s200) and completed (s900–s1200) CCA models showed distinct characteristics from those of the distribution curve produced from TEM images. This tendency is the same as that of the result obtained from the size distribution of NRO clusters.

The other finding is that one of our models can reproduce a similar size distribution of NRO clusters, which might mean that the size distribution is not a critical factor of the incompatibility between our models and the physiological environment.

However, the transition of size distribution of the operating range suggested the existence of a saturation point to produce an operating range partitioned with NRO clusters. One possibility to bear a saturation point for partitioning a two-dimensional space is the balance between the perimeter and the area of the produced partitions.

Next, we compared the transition of size distribution of operating range with the perimeter-to-area ratio of NRO clusters of CCA models at each step to clarify whether these two values affect each other, or whether another factor is necessary to explain the phenomenon.

### 3.3 Perimeter-to-area ratio of the operating range for a mobile particle

**Figure 5:**
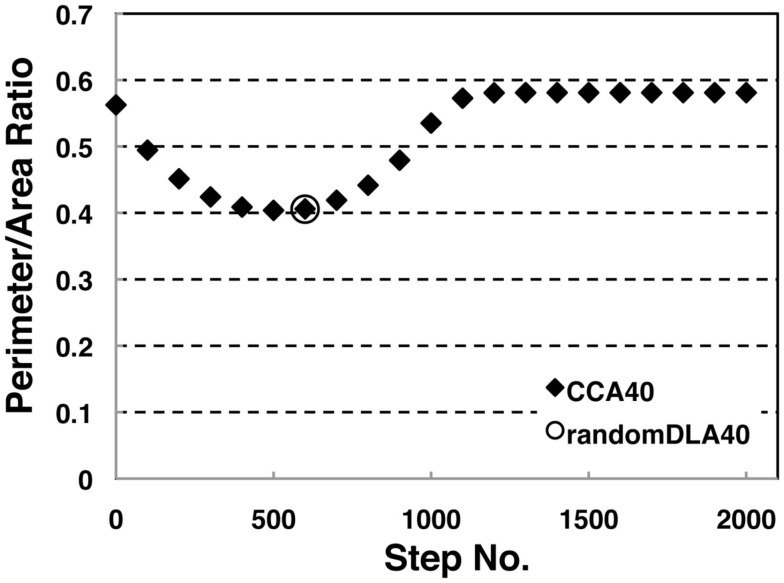
**Perimeter-area ratio of random DLA (40%) and CCA_40_ models**. The *x*-axis shows a step number to build CCA models, and the *y*-axis shows the value of a ratio between the perimeter of whole NRO clusters and the sum of the area occupied by NRO. The bottom value appeared at CCA_40_, s500 (=0.404).

We investigated the perimeter-to-area ratio of the operating range of random DLA (40%) and CCA_40_ models at each step. Results show that the perimeter to area ratio of the operating range in CCA_40_ achieves the smallest value at the 500th step (Figure 5). At the same time, the largest mode of the operating range among CCA_40_ at the different steps appeared at the 500th step (Figures 3C,D). This result shows that these two values have a positive mutual correlation.

**Figure 6:**
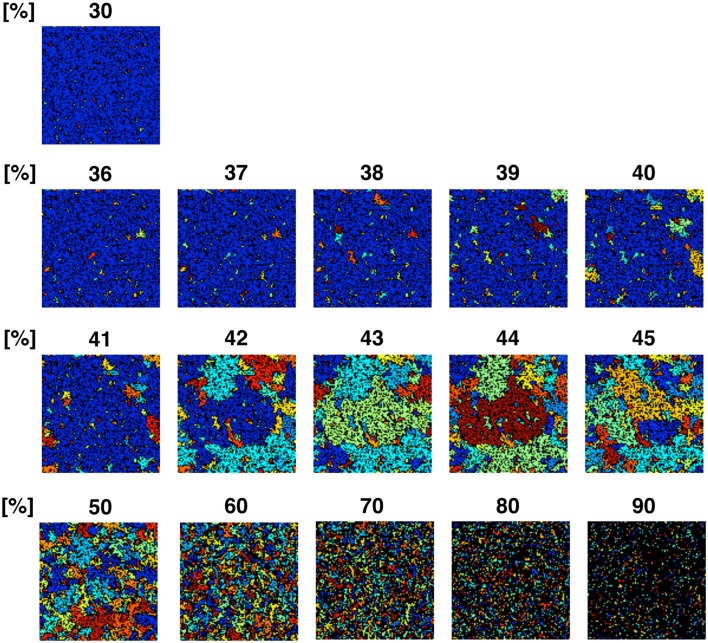
**Partition organizing process during addition of NRO into the reaction space**. The value on the top of each panel shows the relative amount of NRO in the indicated reaction space. Large, homogeneous size partitions appeared among the spaces with 42–45% NRO. With more or less occupied by NRO, the major part of partitions consists of small and huge size spaces.

To confirm the correlation between the perimeter-to-area ratio and the size distribution of the operating range, we simulated the processes of partitioning of a random NRO model with varied relative NRO amounts (Figure 6). In the cases with 43–45% of NRO in reaction space, we observed that the whole reaction space was covered with a similar size of the operating range. At the same time, if the NRO amount is smaller or larger than it is, then the reaction space is occupied by a small number of large operating range or larger NRO cluster. This simulation result is compatible with that when the mode of the operating range expands to the theoretical limiting value, the simulation space is partitioned with a minimum perimeter-to-area ratio. In the sense of a reaction space, too small an operating range cannot function as a reaction space. The reactants will be confined. By including this point, our result shows that the simulation space is covered with the largest sum of the reaction space at the physiological condition. In other words, the physiological environment stands on the most efficient condition to produce the largest sum of the reaction space. The condition can be reconstructed by early CCA_40_, but not by either preliminary completed CCA model in our simulation. The model that can reproduce the physiological environment had a similar size distribution of NRO clusters, and stood on the vicinity of the saturation point of the perimeter-to-area ratio of the operating range. The size distribution was confirmed not to be a critical factor to produce the difference between artificial models and the physiological environment. It must be a side factor because the perimeter-to-area ratio changes according to the analysis target size.

The results presented up to this point show that when models consist of less NRO than in the physiological condition, they can reproduce the same *d_f_* value of NRO clusters with the physiological NRO outline. This incomplete compatibility can occur because of the difference of size distribution of NRO clusters between the physiological environment and the artificial models. However, the size distribution patterns of NRO clusters of CCA models are similar to those of physiological conditions. The distribution of NRO affects the distribution of operation range, which is created by surrounding with NRO clusters. The size of the operating range shows that there exists a saturation point to define the size and the number of the operating range in a simulation space. The saturation point was apparently defined by the perimeter-to-area ratio of the operating range. This result suggests that the physiological intracellular environment stands on the most efficient condition to produce the largest sum of the reaction space. To confirm this hypothesis, we simulated how a reaction space would be partitioned with various relative amounts of NRO. The simulation results show that physiological amounts of NRO divide the simulation space such that the largest sum of the reaction space was produced.

The next question is how a mobile particle in the operating range will behave. The behavior is affected by these environments.

### 3.4 Characteristics of the diffusion molecule in the reconstructed space

In the previous section, we reported that the size distribution of the operating range was most similar between TEM images and CCA with 40% NRO, but before the saturation of distribution (completed CCA) and not in an earlier stage (preliminary CCA).

Next we investigate the MSD of a mobile particle in each model and compare them to clarify the effects of characteristics of these environments. We tested the behaviors of a mobile particle in 1) rNRO, 2) rDLA, and 3) completed CCA. We retained the same conditions at the following eight points for these simulations:

1. The simulation space size was 100 × 100.
2. The simulation space had a periodic boundary condition.
3. We used Euclidean distance to calculate the migration length.
4. The duration of each simulation was 10^4^ steps.
5. We repeated the same test 5,000 times for each condition.
6. At the beginning of the simulation, we left the mobile particle in the model environment randomly.
7. To move this particle, we chose one of right/left/up or down randomly.
8. The mobile particle stopped moving on that step if there was an NRO in the lattice which was chosen as the mobile particle destination.

**Figure 7:**
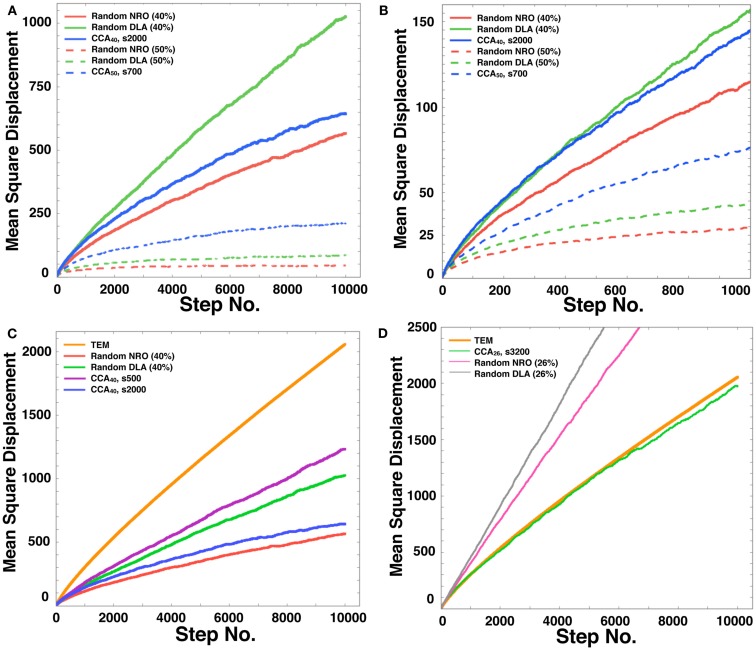
**Mean square displacement of a mobile particle in each model**. **(A)** Mean square displacement of mobile particle diffusion in each model space during a long simulation time up to the 10,000th step (random NRO with 40% of NRO, solid red line; random DLA with 40% of NRO, solid green line; CCA with 40% of NRO at the 2,000th step, solid blue line; random NRO with 50% of NRO, dashed red line; random DLA with 50% of NRO, dashed green line; CCA with 50% of NRO at the 2,000th step, dashed blue line). **(B)** Mean square displacement of mobile particle diffusion in each model space during a short simulation time up to the 1,000th step (random NRO with 40% of NRO, solid red line; random DLA with 40% of NRO, solid green line; CCA with 40% of NRO at the 2,000th step, solid blue line; random NRO with 50% of NRO, dashed red line; random DLA with 50% of NRO, dashed green line; CCA with 50% of NRO at the 2,000th step, dashed blue line). **(C)** Mean square displacement of a mobile particle diffusion in each model space during a long simulation time up to the 10,000th step (TEM image, orange line; random NRO with 40% of NRO, red line; random DLA with 40% of NRO, green line; CCA with 40% of NRO at the 2,000th step, blue line; CCA with 40% of NRO at the 500th step, purple line). **(D)** Mean square displacement of a mobile particle diffusion in each model space during a long simulation time up to the 10,000th step (TEM image, bold orange line; random NRO with 26% of NRO, fine magenta line; random DLA with 26% of NRO, fine gray line; CCA with 26% of NRO at the 3,200th step, fine green line).

For our tests, we chose the case in which 40 or 50% of relative volume of NRO was involved in the simulation space. As explained already, *p_c_ *= 0.593, which means that if the relative volume of NRO is greater than 40.7%, then the operating range will be divided with NRO. Figure 7 presents the respective results obtained using the rNRO, rDLA, and CCA models with 40 (solid lines) or 50% (dashed lines) of NRO. These results show an interesting difference of the characteristics between the 40% NRO and 50% NRO simulation space.

In the 40% NRO space, rDLA had the largest MSD among the three models. However, CCA had the largest MSD among the three models in 50% NRO space. This result might be explained if the NRO relative amount is more or less than the *p_c_*. In general, CCA models produce the largest aggregation of NRO among the three models. Therefore, the perimeter-to-area ratio of NRO is smallest in the CCA model. Consequently, the probability of collision between a mobile particle and NRO is smallest in CCA. This condition was well shown in the results of 50% NRO space. However, when the relative volume of NRO in the space was less than *p_c_*, it is rare for the simulation space to be partitioned completely with NRO in the rNRO and rDLA models. However, the completed CCA model partition simulation space with less relative volume of NRO than *p_c_*. This difference among the three models engendered the results described above. In the 40% NRO space, rDLA was estimated to produce larger MSD than the CCA model. To confirm our hypothesis, we investigated details of the short duration of our simulation. When the simulation time was sufficiently short, the MSD of a mobile particles was larger in the CCA model, and the MSD values were crossed with the rDLA model during the longer simulation time (Figure 7B). The results show that the mobile particle with CCA model is confined in a partitioned operating range.

The results presented in the previous sections show that the characteristics of the simulation space with completed CCA, preliminary CCA, and early CCA are different. The effect on a mobile particle in those simulations spaces might be different. Especially, early CCA shows good compatibility with the physiological environment in the plural points. Another aspect of the characteristics of early CCA among CCA models is that they show the smallest values of the perimeter-to-area ratio, which is the key to changing the MSD value. Therefore, we also simulated MSD of a mobile particle in the early CCA models (s300–s700; Figure 7C presents the result of the 500th step case). In these simulation spaces, the MSD of a mobile particle is larger than that in rDLA with 40% NRO. This result differs for the result of completed CCA (Figures 7A,B). A particle in the preliminary CCA must show larger MSD than the other two CCA. Whether the mobile particle is confined or not is an important point related to obtaining larger MSD. Figure A1 in Appendix depicts that a mobile particle in the preliminary CCA shows smaller MSD than that of the early CCA. These results suggest that the perimeter-to-area ratio is important for a mobile particle to change its MSD.

We compared the CCA_40_, s500 case with the result of a mobile particle in a physiological environment. The MSD of CCA_40_, s500 was the largest among the MSD of artificial models with 40% NRO. However, the MSD remained smaller than the MSD of a mobile particle in a physiological environment. We tested whether the NRO amount is the same condition with which we succeeded in reproducing the close *d_f_* with physiological environment (=NRO 26%; Figure 2, the CCA model can reconstruct a similar MSD value with physiological conditions. The simulation result demonstrated that completed CCA_26_ shows good compatible fluctuation of MSD with physiological conditions (Figure 7D).

These results suggest that the perimeter-to-area ratio is effective for the behavior of a mobile particle. At the same time, the physiological environment consists of more large-area – short-perimeter obstacles compared with the NRO clusters produced by the CCA algorithm. Because of the difference of the shape of NRO clusters and solid structures in a cell, less amount of NRO is required in the simulation space to reproduce the same MSD with physiological conditions by the CCA model. Based on the results described above, we calculated the self-diffusion coefficient of the mobile particle in the three models.

**Figure 8:**
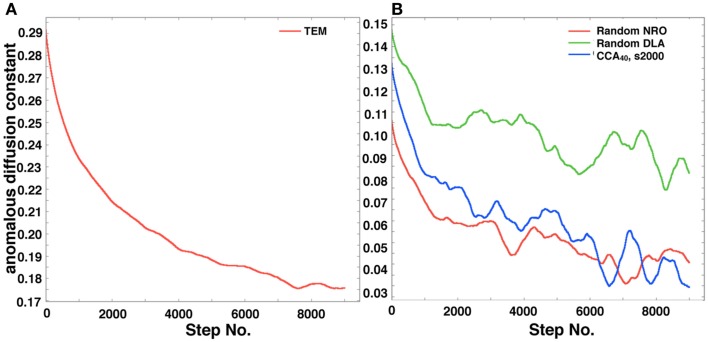
**Anomalous diffusion constant of a mobile particle in a physiological condition or each simulated model**. **(A)** Anomalous diffusion constant of a mobile particle in physiological conditions. A mobile particle was left in a reconstructed environment of the TEM image, and the anomalous diffusion constant was calculated from its MSD. **(B)** Anomalous diffusion constant of a mobile particle in each simulated model (random NRO, red; random DLA, green; CCA with 40% of NRO at the 2,000th step, blue).

Figure 8A shows the self-diffusion coefficient of a mobile particle in physiological conditions. Figure 8B shows the self-diffusion coefficient of a mobile particle in artificial models. These results correlate with the MSD results. The results suggest that the same effect of environment with the case of MSD is observed, which means that the diffusive behavior of a mobile particle in a reaction space is affected by the perimeter-to-area ratio, and that the effect of that physiological environment consists of large-area – short-perimeter clusters than our artificial models was also observed in results of this analysis.

As shown in the results, the MSD value directly changes the diffusive behavior of a particle and affects the reaction process with the molecule. Our results presented herein suggest that the perimeter-to-area ratio of the reaction space is important for more varied behaviors of reactants in an *in vivo* environment.

## 4 Discussion

In our previously reported studies (Hiroi et al., 2011a,b), the operating range was found to be a similar environment with that of the IP model (Wilkinson and Willemsen, 1983). Herein, we described a reconstructed operating range as the free spaces between NRO clusters of our fractal models instead of building an IP model separately because the operating range does exist as the fluid space between solid structures. We tested whether the principle can be true with the fractal models, which we predicted in our previous work (DLA or CCA) as candidates to represent a physiological environment.

As shown in Figure 2, this idea was unable to reproduce an exact environment for a mobile particle that has the same *d_f_* built by the same amount of NRO with the physiological conditions in the simulation space.

We addressed the critical difference between the physiological conditions represented in TEM images and our models: the distribution of the NRO cluster shapes.

The shape distribution can be reduced into two factors. One is the distribution of the size of operating range (Figures 3C,D), and each shape of the NRO cluster shown in Figure 1A and the other panels as their differences. These two parameters were confirmed because they affect the behaviors of a mobile particle in the reaction space (Figures 7 and 8).

Both the size distribution of operating range and the shape of NRO are reasonably translated into the values of the perimeter-to-area ratio of the operating range. The ratio correlated with MSD and diffusion property of a mobile particle in those reaction spaces.

Based on the partitioning simulation with NRO (Figure 6), we estimated that the intracellular environment is partitioned because it can produce the maximum sum of the reaction space.

**Figure A3:**
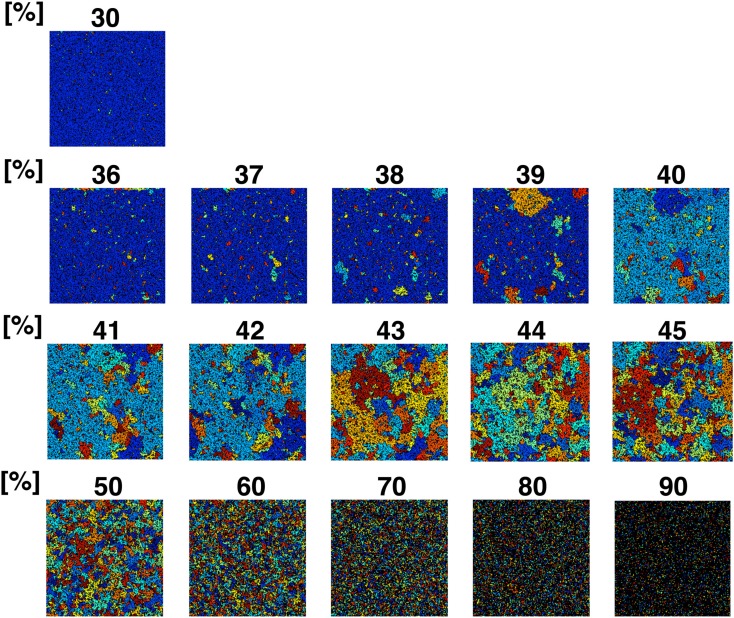
**Partition organizing process during addition of NRO into the reaction space**. The value on the top of each panel shows the relative amount of NRO in the indicated reaction space. Large, homogeneous size partitions appeared among the spaces with 40–45% NRO. With more or less occupied by NRO, the major part of partitions consist of many small partitions and one huge partition.

The simulation result shown in Figure 6 reflects that when the relative amount of the solid structure in a simulation space is near the physiological level (41.36%), the simulation space starts to be fully partitioned as an independent, similar size operating range. This phenomenon is more readily apparent when we use a larger simulation space (Figure A3 in Appendix). The simulation space of the panels in Figure A3 in Appendix was 200 × 200. The transition point appeared when the relative amount of NRO was 40–45% in those spaces. Both the percolation threshold (*p_c_*, 40.7%) and the physiological NRO amount estimated from TEM images (41.36%) were included in these conditions. This results suggest that a physiological amount of crowding molecules is naturally defined as the solid structures that can partition the intracellular space to produce the most efficient pattern of operating ranges for mobile particles.

This property of the environment is compatible with the estimated characteristics of the behavior of intracellular molecules in our previous studies. The MSD *in vivo* is larger than that *in vitro* at the beginning of the reaction. Subsequently, the molecule *in vivo* starts to show confined-molecular-like behaviors. The partitioned environment with a physiological amount of NRO is a sufficient condition to produce such molecular behaviors. Moreover, we confirmed the quick movement following confined-molecular behavior in our simulation (Figure 7B). The result demonstrated that when a simulation space is fully partitioned with NRO, the mobile particle behaves as we estimated.

Our results obtained in these analyses are compatible with basic biological knowledge. Examples of the branch-like obstacles in physiological environment are cytoskeletal proteins. Based on the measured human cell weight and the physiological concentrations of cytoskeletal proteins (Sallusto et al., 1995; Takiguchi et al., 1999; Park et al., 2008), their estimated relative amounts are less than 1% of the total cellular weight. The intracellular environment consists more of the other kind of organelle. Some of them have a lower surface–volume ratio than chain–shaped protein polymers such as lysosomes (sphere), which appear as circles in two-dimensional images, or larger structures, which also have a smaller surface–volume ratio than fine structures have.

In conclusion, our results suggest that a physiological intracellular environment can be defined naturally to produce the largest reaction space for mobile particles with a necessary amount of solid structures. Our simulation demonstrated that the amount of the intracellular solid structure is close to the saturation point to produce the maximum sum of the reaction space in a whole simulation space.

We used a two-dimensional space for our analyses in this paper. Therefore, the characteristics of the intracellular environment appeared as the perimeter-to-area ratio of the operating range. When we examine the same issue in three-dimensional space, the parameter we should investigate will be the surface-to-volume ratio, as we have described.

A well-known hypothesis holds that the size of a cell is defined by a surface-to-volume ratio because a cell can circulate all the necessary biochemical reactions in the volume with the provided source via its surface as a metabolic compartment (Schmidt-Nielsen, 1984).

Now we face two thresholds represented as ratios of particular sets of biological measurements. One is the surface-to-volume ratio of a cell which can define the cell size. When the volume of a cell is too small, a cell cannot perform all the necessary functions for biochemical metabolism. This case is that of a large surface-to-volume ratio. If a cell volume is too large, then the cell cannot be provided sufficient materials for the metabolism. This case is that of a small surface-to-volume ratio.

The other is the surface-to-volume ratio of the reaction space between the solid structures in a cell, which can define the intracellular crowding level. When the crowding level is too low, the intracellular environment works equivalently as an *in vitro* environment with high viscosity. Reactions in such an environment will not be able to respond quickly to the change of the outside of the compartment, or will fail to keep a reaction source by avoiding their exhaustion. This case is that of a small surface-to-volume ratio. If the crowding level is too high, then the intracellular environment is partitioned into many small compartments. The cell cannot complete the necessary metabolism, and the necessary reactants fail to contact each other. This case is that of a large surface-to-volume ratio. The latter half of these perspectives originated from results of our earlier research (Hiroi et al., 2011a,b) and this paper.

For both cases, the surface-to-volume ratio might be the key parameter. Our simulation results (Figure 6; Figure A3 in Appendix) show the dramatic difference of the environment produced before and after the threshold of the ratio. Especially, the condition of the intracellular environment suggests a change from viscous liquid (with less than 41% of NRO) to a crystallized bio-polymer (ex) black NRO connect with each other from top to bottom and also from left to right in the simulation results with 90% of NRO). The unmistakable point is the fact that the measured physiological condition stands on the line of the threshold, which may mean that the physiological environment is in the vicinity of phase transition from an abiosis phase to another abiosis phase.

To confirm these empirical perspectives, further investigation of the size distribution pattern of operating range and the precise definition of the threshold is necessary in the future.

## Conflict of Interest Statement

The authors declare that the research was conducted in the absence of any commercial or financial relationships that could be construed as a potential conflict of interest.

## Supplementary Material

The Movie S1 for this article can be found online at http://www.frontiersin.org/Fractal_Physiology/10.3389/fphys.2012.00293/abstract

We provide movie data to show the self-organization processes of our model.

Supplementary Movie S1 Self-organization process of CCA with 40% of NRO.

## Figures and Tables

**Figure A4:**
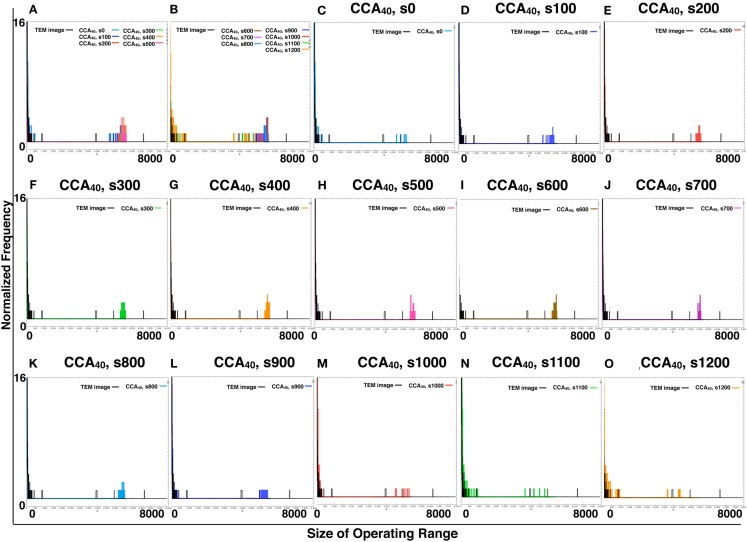
**Comparison of the size of distribution of operating range of TEM image and simulated reaction spaces**. We provide histograms of the size of the operating range of CCA_40_, s0–s1200, made of the same sample number with TEM images (161 images of TEM or 161 images produced by each simulation) to normalize them with the same calculation. **(A,B)** Respectively show the CCA_40_, s0–s500 and s600–s1200, with the size of distribution of operating range of TEM images. The *x*-axes show the size of operating range (0–8000). The *y*-axes show the normalized frequency (0–16). **(C–O)** Showed only two lines in one panel. The size of the distribution of the operating range of one of the models among CCA_40_, s0–s1200, and a TEM image.
